# *In vivo* dosimetry using a single diode for megavoltage photon beam radiotherapy: Implementation and response characterization

**DOI:** 10.1120/jacmp.v2i4.2598

**Published:** 2001-09-01

**Authors:** Valdir C. Colussi, A. Sam Beddar, Timothy J. Kinsella, Claudio H. Sibata

**Affiliations:** ^1^ Department of Radiation Oncology Case Western Reserve University School of Medicine and University Hospitals of Cleveland 11100 Euclid Cleveland Ohio 44106

**Keywords:** in vivo dosimetry, diode detector, radiotherapy, quality assurance

## Abstract

The AAPM Task Group 40 reported that in vivo dosimetry can be used to identify major deviations in treatment delivery in radiation therapy. In this paper, we investigate the feasibility of using one single diode to perform *in vivo* dosimetry in the entire radiotherapeutic energy range regardless of its intrinsic buildup material. The only requirement on diode selection would be to choose a diode with the adequate build up to measure the highest beam energy. We have tested the new diodes from Sun Nuclear Corporation (called QED and ISORAD‐*p*–both *p*‐type) for low‐, intermediate‐, and high‐energy range. We have clinically used both diode types to monitor entrance doses. In general, we found that the dose readings from the ISORAD (*p*‐type) are closer of the dose expected than QED diodes in the clinical setting. In this paper we report on the response of these newly available ISORAD (*p*‐type) diode detectors with respect to certain radiation field parameters such as source‐to‐surface distance, field size, wedge beam modifiers, as well as other parameters that affect detector characteristics (temperature and detector‐beam orientation). We have characterized the response of the high‐energy ISORAD (*p*‐type) diode in the low‐ (1–4 MV), intermediate‐ (6–12 MV), and high‐energy (15–25 MV) range. Our results showed that the total variation of the response of high‐energy ISORAD (*p*‐type) diodes to all the above parameters are within ±5% in most encountered clinical patient treatment setups in the megavoltage photon beam radiotherapy. The usage of the high‐energy buildup diode has the additional benefit of amplifying the response of the diode reading in case the wrong energy is used for patient treatment. In the light of these findings, we have since then switched to using only one single diode type, namely the “red” diode; manufacturer designation of the ISORAD (*p*‐type) high‐energy (15–25 MV) range diode, for all energies in our institution and satellites.

PACS number(s): 87.66.–a, 87.53.–j

## INTRODUCTION

*In vivo* dosimetry has been used to improve the quality of patient care in radiation therapy by verification of external beam treatment fields.^1–^, ^3^ Several methods are currently available for this use such as thermoluminescent dosimeters (TLD), Metal oxide‐silicon semiconductor field effect transistors, and semiconductor diodes.^4–^, ^7^ TLDs have been the more commonly used, however, these systems are labor intensive, which makes them impractical for use on every patient. In most cases, TLD's are only used for special treatment procedures such as total body irradiation, total skin electron irradiation, and unusual treatment configurations or to monitor doses to critical structures. Silicon diode detectors have gained popularity as *in vivo* dosimeters because they provide a convenient way of measuring the patient entrance doses in real time and are easier to use by the therapists.^3^ ^,^ ^8^ The American Association of Physicist in Medicine has reported on using diodes for *in vivo* dosimetry.^9^

It is known that the diode response varies significantly with the treatment beam setup.^2,^, ^6^ It has been found that it is necessary to apply additional correction factors to take into account their response as a function of source‐to‐surface distance (SSD), field size, wedged field, and diode orientation.^2,^, ^7,^, ^10^

The commercial Sun Nuclear Corporation (Melborne‐Fl–http://www.sunnuclear.com) diode detectors that are presently available on the market for photon *in vivo* dosimetry are ISORAD (*n*‐type), QED (*p*‐type), and ISORAD (*p*‐type) detectors (listed in the chronological order of their commercialization). Zhu^10^ recently characterized the response of the commercial ISORAD (*n*‐type) and the QED (*p*‐type) diode detectors. Zhu has studied the SSD, field size, and wedge‐dependence for those two types of diode detectors for *in vivo* dosimetry, and found that in general, the correction factors for the ISORAD (*n*‐type) diode detectors are larger than the QED (*p*‐type) diode detectors. In this paper, we are studying the SSD, field size, wedge, beam orientation (angular‐dependence), temperature‐dependence of the ISORAD (*p*‐type) diodes, as well as comparing them to the QED (*p*‐type) diode detectors. The newly available ISORAD (*p*‐type) diode detectors, which became available in the market since 1999, are an improved version of the original ISORAD detectors. The original ISORAD detectors are *n*‐type silicon *p‐n* junction diodes and have been available on the market within the last few years (5–6 years). The cylindrical configuration and buildup material is the same for both diodes except that the detector element for the newly available diodes has been replaced with a new proprietary *p*‐type silicon diode. Furthermore, the ISORAD (*p*‐type) detectors have an improved radiation resistance when compared to the original ISORAD (*n*‐type) detectors.

## MATERIALS AND METHODS

The ISORAD (*p*‐type) detector series consist of four photon detectors. There are three detectors covering the following ranges: 1–4 MV (blue diode), 6–12 MV (gold diode), and 15–25 MV (red diode). The fourth detector (black diode) in the series is a specially compensated detector designed for lower photon energies (e.g., scattering). The general specifications of these diode detectors are available from the manufacturer (Sun Nuclear). In this study we have investigated the three above mentioned detectors to cover the entire megavoltage photon beam radiotherapy (1–25 MV), using each detector in its corresponding energy range as recommended by the manufacturer. In addition the red diode, which is specifically designed for 15–25 MV, was tested for the entire megavoltage range, since the purpose of this work is to investigate the feasibility of using one diode regardless of photon energy. The only requirement on the diode to be selected would be to choose a diode with enough buildup so that the point of measurement will be beyond the buildup region [in other words, in the charge particle equilibrium (CPE) or transient CPE region]^11^ and also to eliminate most of the electron contamination produced by photon beams. For instance, if one uses a diode with a low‐energy buildup to measure a 18‐MV photon beam, then the measurement will occur in the buildup region and would be extremely susceptible to slight changes in the geometrical conditions of the irradiation (tangential fields, curved surfaces, etc.). Therefore, we selected a diode that would have enough buildup to measure the highest photon beam energy in the radiotherapy range (25‐MV photon beam), which corresponds to the “red diode.” We will use the color code chosen by the manufacturer to refer to the high‐energy range diode (15–25 MV) in this paper.

As mentioned in the Introduction, we also compared the ISORAD (*p*‐type) diode detectors to the QED (*p*‐type) diode detectors. The QED detector series consist of three photon detectors each for a different energy range: 1–4 MV (blue diode), 6–12 MV (gold diode), and 15–25 MV (red diode). Note that the manufacturer kept consistently the same energy range as well as the color coding for the newly manufactured ISORAD (*p*‐type) diodes as the QED diodes. In this paper we will interchangeably use either the color code or the energy range to designate these diodes.

The *x*‐ray beam energies used in this study were 4 MV (Clinac 4/100–Varian Medical System, Inc., Palo Alto, CA), 6 and 10 MV (Mevatron 6–10–SiemensMedical Laboratories, Inc., Walnut Creek, CA), 6 and 18 MV (Clinac 2100 C/D–Varian Medical System, Inc., Palo Alto, CA). All photon beams were calibrated using isocentric setup according to the AAPM Task Group 51 protocol,^1,^, ^2^ and were calibrated to deliver 1.00 cGy/MU in muscle for a $10\times 10\;{\text{cm}}^{2}$ field size at the depth of maximum buildup ($d_{\max}$).

**Figure 1:**
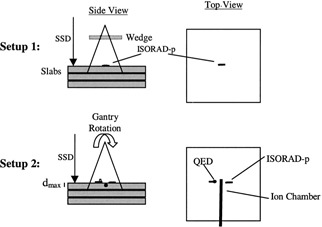
Schematic diagram showing views of the setup used to evaluate the diode dosimetry response.

In this paper we define “diode correction factors” (DF) the ratio of the dose measured by the diode and the expected dose for the given condition. In order to determine diode correction factors for wedge (${\text{DF}}_{\text{Wedge}}$), Field Size (${\text{DF}}_{\text{FieldSize}}$), and SSD (${\text{DF}}_{\text{SSD}}$), a systematic procedure was developed. Measurements were performed under a variety of clinically relevant configurations: open square fields, square fields with different wedges, and source‐to‐surface distances (SSDs) ranging from 80–140 cm, and collimator settings ranging from $4\times 4\;\text{to}\;40\times 40\;{\text{cm}}^{2}$ field size. ${\text{DF}}_{\text{Field Size}}$ were obtained by normalizing the diode wedged field size readings with its own machine wedged field size factors and further normalized with the reading to the $10\times 10\;{\text{cm}}^{2}$ field size at a 100‐cm SSD. Diode SSD factors (${\text{DF}}_{\text{SSD}}$) were obtained by normalizing the diode wedged SSD readings with its own machine wedge and by the inverse square factor and further normalizing with the reading to the $10\times 10\;{\text{cm}}^{2}$ field size at a 100‐cm SSD. For experiments using only a single diode, the diode detector was placed on the central axis of the beam as shown in Fig. 1 (Setup 1). In the experiments using a pair of detectors, each detector was placed 2 cm from the central axis, so that both detectors will be measuring simultaneously the radiation beam as illustrated in Fig. 1 (Setup 2).

The diode angular‐dependence was measured by rotating the gantry over the diode that was placed at the machine isocenter Fig. 1 (Setup 2). The angular diode factor (${\text{DF}}_{\text{angle}}$) was obtained normalizing their reading with the reading obtained at normal incidence (Gantry at 0° degree).

The stability of the QED diodes (blue, gold, and red) and the red ISORAD diodes were determined by exposing these diodes to their corresponding energy range (low‐, intermediate‐, and high‐energy range for the QED's and all three ranges for the red ISORAD diodes) using Setup 2 as shown in Fig. 1.

**Figure 2:**
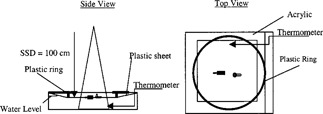
Thermal water phantom.

The thermal diode correction factor (${\text{DF}}_{\text{Temperature}}$) was measured using a small water tank covered with a thin plastic cover sheet. The diodes were placed on this cover sheet, which was forced to touch the water bath using a plastic ring as illustrated in Fig. 2. The temperature was carefully monitored using a waterproof thermocouple digital thermometer (Digital Thermometer 500–VWR Scientific San Francisco).

The attenuation of the beam was evaluated using film dosimetry. Kodak *X*‐Omat‐V films placed perpendicular to the beam were irradiated at the depth of 0 cm (surface), 5 cm, and 10 cm in a polystyrene phantom.^13,^, ^14^ The ${\text{ISORAD}}_{\text{red}}$ (*p*‐type) diode was placed at the central axis for a $10\times 10\;{\text{cm}}^{2}$ field at 100‐cm SSD. The films within the above phantom configuration were exposed using 4, 6, and 18 MV *x*‐ray beams. The variation of the optical density on the irradiated film induced by the buildup material cap of the ${\text{ISORAD}}_{\text{red}}$ was evaluated using a VIDAR scanner and a film dosimetry scanning system (RIT™).

## CLINICAL MODEL

In the clinical setup, the calibrated diode is placed in the center of the treatment field. The “entrance dose” ($D_{\text{Entrance}}$) is defined as the dose at depth of maximum dose for the corresponding energy. The diode reading that is expected for each treatment field is given by

$$R_{\text{Expected}}=\frac{D_{\text{Entrance}}}{DF_{i}},$$

where ${\text{DF}}_{i}$ are the individual diode correction factors taking into account nonreference conditions (${\text{DF}}_{\text{Wedge}}$, ${\text{DF}}_{\text{FieldSize}}$, ${\text{DF}}_{\text{SSD}}$, ${\text{DF}}_{\text{etc}}$). The definition of these is consistent with previously published data.^10^ In this paper, ${\text{DF}}_{\text{FieldSize}}$ are assumed to be independent of ${\text{DF}}_{\text{SSD}}$ and ${\text{DF}}_{\text{SSD}}$ are assumed to be independent of ${\text{DF}}_{\text{FieldSize}}$.^14^

For calibration, the ISORAD (*p*‐type) for high‐energy buildup diodes (red), were placed on the central axis of the solid phantom for a $10\times 10\;{\text{cm}}^{2}$ field size at a 100‐cm SSD (reference condition) as shown in Fig. 1 (Setup 1). Two hundred monitor units were delivered for each calibration exposure, which was related to the dose at $d_{\max}$ for each specific energy. Therefore, for each energy, the diode reading is related to an entrance dose in the reference condition. Calibration checks are performed every month as part of our quality assurance program and adjustments, if necessary, are made for the reference condition.

## RESULTS AND DISCUSSIONS

### A. Initial testing and reproducibility

The stability of the QED diodes (blue, gold, and red) and the red ISORAD diodes were determined by exposing these diodes to their corresponding energy range (low‐, intermediate‐, and high‐energy range for the QED's and all three ranges for the red ISORAD diodes) using Setup 2 as shown in Fig. 1. The diode readings were normalized to the first measurement. The reproducibility of the diode readings resulted in a standard deviation equal to 0.001 from ten consecutive measurements with all measurements being within ±0.1% of the first reading. However, measuring the diode response daily for the first ten days and weekly for the following ten weeks resulted in maximum variation of ±1% from the first measurement with a standard deviation equal to 0.001. A monitoring ionization chamber was used to prevent accelerator daily output variations from influencing the results of these tests.

### B. Field size factors

#### 1. Diodes open field correction factors

**Figure 3:**
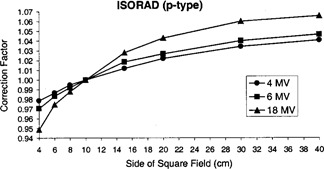
ISORAD (*p*‐type) diode open field size correction factors for 4, 6, and 18‐MV photon beams for 100 SSD.

The diode open field size correction factors (${\text{DF}}_{\text{FieldSize}}$) for the high‐energy ISORAD (*p*‐type) diode are shown in Fig. 3 for 4, 6, and 18‐MV photon beams. These diode correction factors are quite different from the field size output factors of the linear accelerator (i.e., total scatter factors) for all energies. We also have found out that the diode open field correction factors for ISORAD (*p*‐type) diodes are larger than those of the QED diodes as was previously shown by Zhu.^10^ These results are shown in Table I. Zhu compared QED diodes to ISORAD (*n*‐type), whereas in this table we are comparing QED diodes to the ISORAD (*p*‐type). Table I also shows that the open field correction factors of the ISORAD (*p*‐type) and the ISORAD (*n*‐type) are almost identical.

**Table I:** Diode open field size correction factors, comparison between QED and ISORAD (p‐type).

	4 MV	18 MV		4 MV	18 MV	
Field Size	This Work		Zhu's Work	This Work		Zhu's Work
	${\text{QED}}_{\text{Red}}$ (*p*‐type)	${\text{QED}}_{\text{Red}}$ (*p*‐type)	QED (*p*‐type)	${\text{ISORAD}}_{\text{Red}}$ (*p*‐type)	${\text{ISORAD}}_{\text{Red}}$ (*p*‐type)	ISORAD (*n*‐type)
4	0.993	0.970	0.97	0.978	0.949	0.95
6	0.998	0.988	0.99	0.987	0.974	0.97
8	0.999	0.993	1.00	0.995	0.988	0.99
10	1.000	1.000	1.00	1.000	1.000	1.00
15	1.000	1.013	1.02	1.012	1.028	1.02
20	1.000	1.018	1.03	1.022	1.043	1.04
30	1.000	1.024	1.03	1.034	1.060	1.06
40	1.000	1.024	1.04	1.041	1.066	1.06

### C. Diode wedged field correction factors

Diode wedge correction factors (${\text{DF}}_{\text{Wedge}}$) were measured for the blue, gold, and red QED diodes as well as for the red ISORAD (*p*‐type) diode for 4, 6, and 18‐MV photon beams. These measurements are listed in Table II, where we compare the red ISORAD (*p*‐type) diodes to the QED diodes in the respective energy range.

**Table II:** Comparison of diode wedge correction factors for QED (p‐type) in their respective energy and ISORAD (p‐type) for 4, 6, and 18‐MV photon beams with the field size of $10\times 10\;{\text{cm}}^{2}$ at 100 SSD.

	4 MV		6 MV		18 MV	
Wedge	QED (*p*‐type) (Blue)	ISORAD (*p*‐type) (Red)	QED (*p*‐type) (Gold)	ISORAD (*p*‐type) (Red)	QED (*p*‐type) (Red)	ISORAD (*p*‐type) (Red)
15°	1.005	1.000	1.017	0.998	1.15	1.004
30°	1.009	0.997	1.022	1.000	1.016	1.004
45°	1.018	0.993	1.038	1.001	1.029	1.041
60°	1.026	0.983	1.060	1.013	1.040	1.052

Our data has shown that the diode wedge factors for the same ${\text{ISORAD}}_{\text{Red}}$ (*p*‐type) for 4, 6, and 18 MV are in general smaller than the diode wedge factors for QED (*p*‐type). The high‐energy buildup material seems to reduce the instantaneous dose rate‐dependence for lower energies (<6MV). This effect may be explained by the variation of electron contamination in the buildup region.^14^

### D. Diode SSD correction factors

Table III shows the equivalence of the diode SSD correction factors (${\text{DF}}_{\text{SSD}}$) for red QED (*p*‐type) and red ISORAD (*p*‐type) when they were exposed to the low (4 MV) and high (18 MV) energy photon beam. This table also shows the diode correction factor for the blue QED (*p*‐type) and red ISORAD (*p*‐type) for both exposed to low‐energy photon beams (4 MV). The ${\text{DF}}_{\text{SSD}}$ for ISORAD (*p*‐type) at low‐energy seems to be similar to the blue QED (*p*‐type).

**Table III:** Comparison of the diode SSD correction factors for both the QED and the ISORAD (p‐type) diodes for SSD's ranging from 80 cm to 140 cm for $10\times 10\;{\text{cm}}^{2}$ field size.

	4 MV		18 MV	
SSD	${\text{QED}}_{\text{Blue}}$ (*p*‐type)	${\text{ISORAD}}_{\text{Red}}$ (*p*‐type)	${\text{QED}}_{\text{Red}}$ (*p*‐type)	${\text{ISORAD}}_{\text{Red}}$ (*p*‐type)
80	0.983	0.998	0.973	0.970
90	0.985	0.999	0.987	0.989
100	1.000	1.000	1.000	1.000
110	1.002	1.001	1.010	1.011
120	1.005	1.002	1.020	1.020
130	1.009	1.003	1.027	1.029
140	1.010	1.004	1.035	1.035

### E. Diode angular correction factor

**Figure 4:**
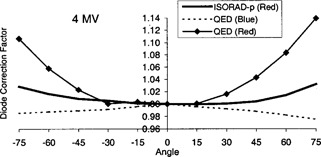
Diodes angular correction factor.

An important source of variance using QED diodes is its response for angled fields. Treatment fields such as tangential fields for breast radiation are subjected to incident beam not perpendicular with the surface of the diode. In order to evaluate this contribution quantitatively, the response of diodes as a function of the Gantry position was evaluated at various angles (−75° to +75°) using Setup 2 (Fig. 1). The diode readings were normalized to the diode reading obtained at 0° gantry angle for a $10\times 10\;{\text{cm}}^{2}$ field size, and an SSD equal to a 100 cm. The data show that the QED series diode readings have a significant angular‐dependence that needs to be taken into account. As expected, the ISORAD (*p*‐type) diodes show a minor angular‐dependence due to their cylindrical shape. Although the measured data for these cylindrical diodes show a small dependence at large angles. The results obtained when exposing these diodes to a 4‐MV photon beam are shown in Fig. 4. When using the QED diodes, the above results were used to correct the diode angular‐dependence (${\text{DF}}_{\text{angle}}$). Whereas no corrections were needed when using the ISORAD (*p*‐type) diodes.

### F. Diode thermal‐time correction factor

The sensitivity variation with temperature of dosimetry diodes is well‐known.^15–^, ^17^ The temperature‐dependence in *p*‐type silicon detectors have also been well described in the literature,^15,^, ^18^ and therefore, will not be discussed further.

**Figure 5:**
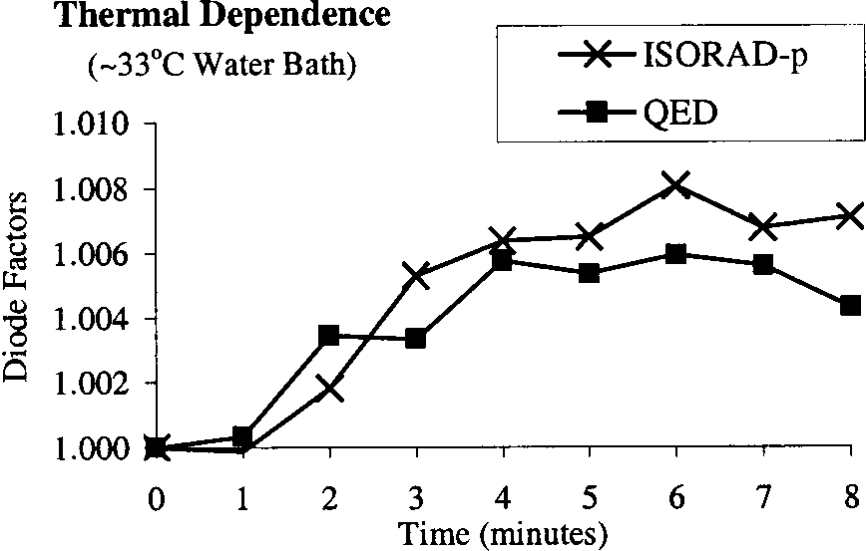
Diodes thermal correction factor.

The diode response variation over time to normal body temperature exposure was evaluated using a $10\times 10\;{\text{cm}}^{2}$ field size at a 100‐cm SSD. Under normal conditions, the body skin temperature is approximately around 33°C. Therefore, the diodes (QED and ISORAD‐*p*), which were initially at normal room temperature (~22 °C) were placed on the water phantom shown in Fig. 2. The water temperature was raised to a temperature equal to 33 °C and was allowed to stay in thermal equilibrium with the surrounding water bath. This experiment simulates clinically the thermal effect on the diode when it comes in contact with patient skin. In these experimental conditions, the temperature of the water bath is different from the temperature of the diode. Even after the equilibrium, the temperatures might differ from each other. In our clinical setting, the diode does not stay in contact with the patient skin for more than 2 minutes. One hundred Monitor units were delivery from a Clinac 4/100 each minute after the diode was placed on the surface over a period duration of 8 minutes. The readings were normalized with the first reading. Figure 5 shows that an increase of less than 1% is observed from the initial time at which the diode was at room temperature. Therefore, based on our experimental results we concluded that for these diode series and under those experimental and clinical conditions, the temperature correction factors are negligible.

### G. Dose perturbation due to diodes

The buildup thickness for the ${\text{ISORAD}}_{\text{red}}$ (*p*‐type) is equivalent to $2.58\;g/{\text{cm}}^{2}$ water (Sun Nuclear). The red diode designed for entrance dose will be expected to perturb the dose distribution delivered to the patient as reported in the literature.^13,^, ^14,^, ^19^

Sen *et al.* ^19^ have quantitatively assessed dose perturbation caused by silicon diodes on photon and electron‐beam characteristics. Alecu *et al.* ^13^ have studied in detail the dose perturbation to the patient due *in vivo* dosimetry in photon beams with diodes. The same general findings that have been reported by Sen *et al.* ^19^ and Alecu *et al.* ^13^ would apply to the ISORAD (*p*‐type) diodes. The main difference would be that the dose reduction to the patient when using the red diode at the lower energy beams would be higher than what has been reported in the above earlier work. Because, in this case, we will be using a diode with the high‐energy beam buildup material to monitor lower photon beam energies. To quantify this effect, we followed the same method adopted by Alecu *et al.* ^13^ to measure the dose perturbation due to these high‐energy ISORAD (*p*‐type) diodes. Table IV summarizes these results.

**Table IV:** Dose reduction due to the buildup cap for ${\text{ISORAD}}_{\text{red}}$ (p‐type) for low‐ and high‐energy photon beams.

	ISORADred (*p*‐type)			Alecu's Work
Depth (cm)	4 MV	6 MV	18 MV	15 MV
0	–45%	–50%	–80%	–89.2%
5	30%	25%	12%	13.2%
10	15%	15%	15%	13.0%

The dose reduction is more pronounced than the finding of Alecu *et al.* ^13^ due to the fact that we are using the diode with a higher buildup material for low‐energy photon beams. Our results for the 18‐MV *x*‐ray beam compare fairly well with their data for the 15‐MV *x*‐ray beam as shown in Table IV.

## CONCLUSION

The reproducibility of the diodes (for both QED and ISORAD‐*p*) is good (up to 1%) for clinical purposes. However, a calibration factor of the diode system needs to be checked periodically because of the well‐known damage with the *x*‐ray's energy, which may cause the response of the diode to drift over time.^7^

The angular‐dependence up to 14% for large gantry angles of the QED diode series has been improved by the ISORAD‐*p* series, thus, eliminating the extra care from the radiation therapists.

The attenuation caused by a high‐energy buildup for low and high energy is dependent on the depth. However, for measurements done only at first treatment day, the results represent a dose of less than 2% for standard treatments with five sessions per week.

In summary, we conclude from the results of this work that high‐energy buildup diodes (Sun Nuclear Corporation) can be used for in vivo dosimetry in the entire megavoltage energy range used in radiotherapy.
